# Fatal Pulmonary Hypertension and Right-Sided Congestive Heart Failure in a Kitten Infected with *Aelurostrongylus abstrusus*

**DOI:** 10.3390/ani10122263

**Published:** 2020-12-01

**Authors:** Tommaso Vezzosi, Stefania Perrucci, Francesca Parisi, Simone Morelli, Michela Maestrini, Giulia Mennuni, Donato Traversa, Alessandro Poli

**Affiliations:** 1 Department of Veterinary Sciences, University of Pisa, Viale delle Piagge n. 2, 56124 Pisa, Italy; tommaso.vezzosi86@gmail.com (T.V.); stefania.perrucci@unipi.it (S.P.); francesca.parisi@vet.unipi.it (F.P.); michela.maestrini@phd.unipi.it (M.M.); 2 Faculty of Veterinary Medicine, University of Teramo, Piano d’Accio, 64100 Teramo, Italy; smorelli@unite.it (S.M.); dtraversa@unite.it (D.T.); 3 Studio Associato Veterinario Razzauti Daolio Anguillesi, 57100 Livorno, Italy; giulia_mennuni@yahoo.it

**Keywords:** *Aelurostrongylus abstrusus*, ascites, cardiology, congestive heart failure, domestic cat, echocardiography, parasitic bronchopneumonia, pulmonary hypertension

## Abstract

**Simple Summary:**

Infections caused by lungworms are an emerging issue in feline medicine. Clinical features in cats may vary from subclinical infections to a severe disease, occasionally including fatal pneumonia, depending on different factors, e.g., lungworm species, parasitic burden, and age of the animal. A case of infection caused by *Aelurostrongylus abstrusus* in a domestic kitten presenting acute dyspnoea and ascites is presented here. Clinical, radiological, echocardiographic, parasitological, molecular, and pathological data are described. This is the first report of life-threatening pulmonary hypertension inducing congestive heart failure caused by *A. abstrusus* infection in a domestic kitten.

**Abstract:**

*Aelurostrongylus abstrusus* is considered the most important respiratory nematode of domestic cats worldwide. This parasite inhabits the alveoli, alveolar ducts, and bronchioles and causes a subacute to chronic respiratory clinical disease. Clinical signs may occur in domestic cats of any age, though they are more often described in young animals. Physical examination, echocardiography, thoracic radiography, pulmonary and cardiac pathological findings, classical, and molecular parasitological analysis of a six-month-old kitten referred at the Veterinary Teaching Hospital of the University of Pisa (Italy) led to a diagnosis of parasitic bronchopneumonia caused by *A. abstrusus*, which was complicated by severe pulmonary hypertension (PH) and right-sided congestive heart failure (R-CHF) that caused the death of the animal. Cases of reversible PH associated with *A. abstrusus* infection have been seldom reported in cats. This is the first report of fatal PH and R-CHF in a kitten with clinical aelurostrongylosis.

## 1. Introduction

The cosmopolitan metastrongylid *Aelurostrongylus abstrusus*, known as the “cat lungworm”, is the most prevalent respiratory nematode of the domestic cat [1,2]. This respiratory nematode infects cats of all ages, regardless of their habitat, lifestyle, breed, or gender [2,3]. In Europe, *A. abstrusus* is distributed throughout countries, and in Italy, especially in central-southern regions [4,5]. In Europe, infection rates vary widely from 1.2% in owned to 50% in free roaming cats [2]. In the Americas, *A. abstrusus* has been recorded with rates from 0.08% in client-owned to 5.1% in shelter cats in North America [6,7], and from 0.21% to 39.2% in different domestic cat populations of South America [8].

The life cycle of *A. abstrusus* involves gastropod intermediate hosts, though cats most often become infected preying on small vertebrates, such as mice, birds, reptiles, and amphibians, serving as paratenic hosts [1,2]. The continuous availability of paratenic hosts is the most important reason why cats are at risk of being infected throughout the year [1,3]. Furthermore, kittens may acquire the infection through the ingestion of tissues of preys offered or regurgitated by the queen during the weaning period [1,9].

Adults of *A. abstrusus* infect alveoli, alveolar ducts, and bronchioles of the cat, and cause subacute, acute, or chronic respiratory diseases in cats of any age, although clinical signs are usually more frequent and severe in young subjects [1,2,10,11]. Cough, tachypnoea, and dyspnoea of varying severity, but rarely with a fatal outcome, are the most frequent findings in *A. abstrusus*-infected cats [1,2,3]. In more severe cases, pneumothorax, pleural effusions, and asthma-like clinical signs can be observed [3,11].

The main pathological findings associated with cat aelurostrongylosis are caused by nematode adults, larvae, and eggs, and are represented by multifocal granulomatous and inflammatory cell infiltrates in the pulmonary parenchyma and in the alveoli of peribronchial areas, alveolar wall thickening, and musculature hypertrophy [12,13]. Cell infiltrates in the bronchial walls, presence of mucus and inflammatory cells in the bronchial lumen, and smooth muscle hypertrophy have also been reported [13].

Pulmonary hypertension (PH) in cats has been described in association with congenital heart diseases [14,15,16], pulmonary thromboembolism [17], pulmonary fibrosis [18], chronic upper airway obstruction [19], and left-sided congestive heart failure [20]. PH has been occasionally diagnosed in infected cats, i.e., a single case of irreversible PH in a cat infected by *Troglostrongylus brevior* in Italy [21] and reversible PH in one kitten with aelurostrongylosis in the Netherlands [22]. This study describes the first report of severe and life-threatening PH inducing R-CHF caused by *A. abstrusus* in a domestic kitten.

## 2. Materials and Methods

### 2.1. Clinical Case

A 6-month-old female shorthair kitten, 1 kg in weight, was referred to the Veterinary Teaching Hospital of the University of Pisa, central Italy, for progressive cough, worsening dyspnoea, and dysorexia in the last 5 days. The kitten had mainly an outdoor lifestyle in a countryside environment. Physical examination revealed pink-pale mucous membranes, cachexia, and tachypnoea (55 breaths per minute) with a restrictive breathing pattern. Lung auscultation revealed diffuse pulmonary crackles. Weak femoral pulse was present bilaterally. Heart rate was 180 beats per minute with regular rhythm, and the cardiac auscultation revealed a grade 4/6 right basilar holosystolic murmur. The kitten was mildly hypothermic (rectal temperature, 37.3 °C) and had an abdominal distention. The animal was immediately hospitalized in critical clinical condition. The main differential diagnosis based on history and physical examination included: congestive heart failure, pneumonia, feline infectious peritonitis. Thoracic radiology and echocardiography were performed, and faeces (about 3 g) were collected for parasitological examinations. Based on diagnostic findings (see results section), the cat was diagnosed with parasitic bronchopneumonia with secondary PH and R-CHF. Oxygen therapy was administered through an oxygen cage. The kitten was treated with methylprednisolone 0.5 mg/kg IV q24h, furosemide 1 mg/kg IV q12h, and sildenafil 1 mg/kg PO q12h. Nevertheless, a few hours after hospitalization, the clinical conditions progressively worsened until a sudden cardiorespiratory arrest. Written informed consent was obtained from the owners to perform the necropsy.

### 2.2. Thoracic Radiology and Echocardiography

Chest radiographs were performed using a digital system (FPD 4343A GOS/CSI, Isomedic srl, Lodi, Italy). The respiratory distress of the patient allowed for only lateral views.

Standard echocardiography was performed in a standing position due to the patient’s critical condition. The echocardiographic examination was performed using an ultrasonographic unit (Aplio 300, Canon Medical Systems Europe, Zoetermeer, The Netherlands) equipped with an electronic phased-array transducer (4–9 Mhz). Left chamber dimensions, wall thicknesses, and ventricular function were assessed as previously described [20]. Right-heart dimension and function were subjectively and objectively assessed [20,23,24]. The presence of interventricular septal flattening was subjectively assessed. The dimension of the pulmonary artery was calculated as the ratio between the pulmonary trunk and the aorta, and the pulmonary systolic flow profile was assessed using pulsed wave Doppler [20]. The tricuspid regurgitation was evaluated using colour and continuous wave Doppler. Pulmonary hypertension was defined as a peak tricuspid regurgitation velocity > 2.7 m/s [20].

### 2.3. Parasitological and Molecular Analysis

Faecal samples were microscopically analysed by a conventional flotation assay using a saturated NaCl solution (specific gravity 1.2), and by the Baermann migration test [25]. First stage larvae (L1) recovered at the Baermann test were counted and then identified based on their morphologic and morphometric features [25]. After the post mortem examination (Section 3.2), a portion of a lung was opened, and pulmonary tissue smears were examined for nematode eggs and larvae. Then, the lung piece was washed in saline solution in a conic beaker for 12 h at 4 °C. Then, the pulmonary lavage liquid was microscopically analysed under an inverted and an optical microscope, for the detection and identification of larval nematodes [26]. An aliquot of faeces, Baermann’s sediment, and the lung lavage liquid found positive for larvae in the microscopic examinations were subjected to a previously described molecular test specific for the most common feline metastrongyloids [27]. Briefly, a multiplex PCR method able to simultaneously identify *A. abstrusus*, *T. brevior*, and *Angiostrongylus chabaudi*, and consisting of a first step with universal primers NC1 and NC2 and a second step using diagnostic primers in combination with NC2 to achieve specific amplifications for the three nematodes, was used [27].

### 2.4. Histopathological Methods

Representative tissue samples of stomach, intestine, lymph nodes (peripheral, mesenteric, and mediastinal), spleen, liver, kidney, adrenal glands, ovary, uterus, lung, heart, thymus, encephalon (telencephalon, diencephalon, mesencephalon, pons, cerebellum, and medulla), and spinal cord were fixed in 10% neutral buffered formalin and routinely embedded in paraffin wax. Four micron thick sections were stained with Haematoxylin and Eosin, Goldner’s Trichrome, and Periodic Acid Schiff, Perls, and Toluidine Blue stains.

## 3. Results

### 3.1. Clinical Evaluation

**Figure 1:**
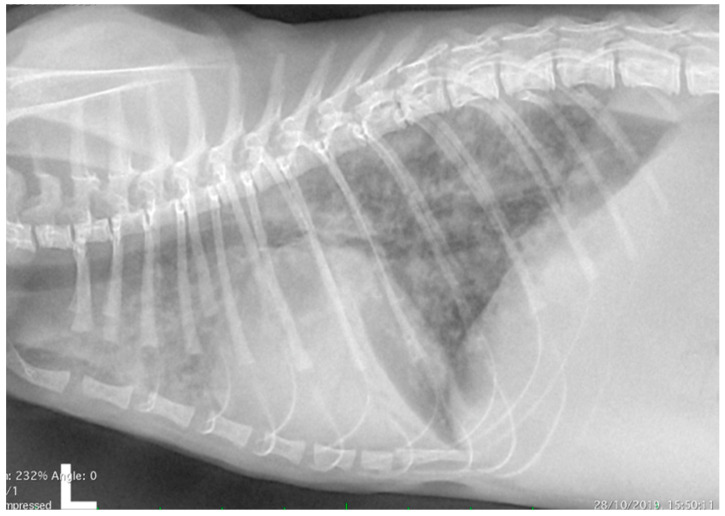
Thoracic radiograph (lateral view) from a 6-month-old domestic kitten with progressive dyspnoea. Diffuse interstitial and alveolar pattern, with mild pleural effusion, was evident. The increased sternal contact of the cardiac silhouette suggested right-side cardiomegaly.

Thoracic radiographs showed a diffuse interstitial and alveolar pattern, with mild pleural effusion (Figure 1). The increased sternal contact of the cardiac silhouette suggested right-side cardiomegaly.

**Figure 2:**
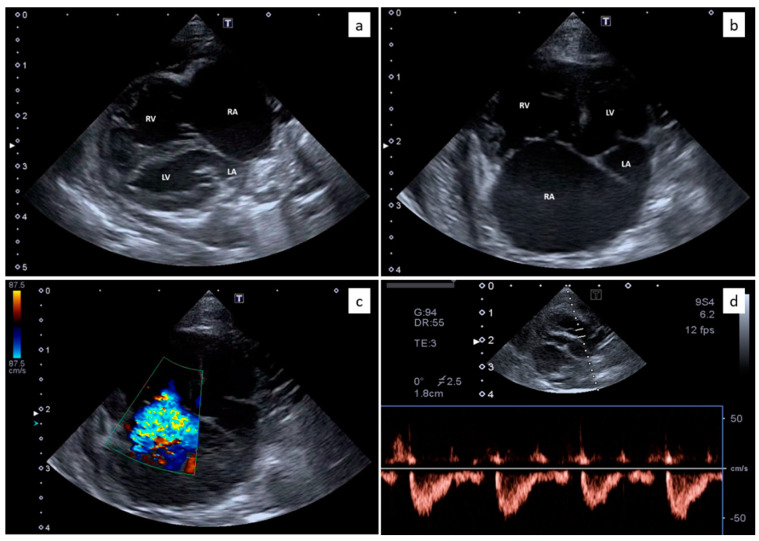
Echocardiography from the domestic kitten showing severe right atrial (RA) and right ventricular (RV) dilation, with reduced dimensions of the left atrium (LA) and the left ventricle (LV) (**a**,**b**). Moderate to severe tricuspid regurgitation was evident (**c**) with normal morphology of the valvular leaflets. The pulmonary artery systolic flow had a proto-systolic peak (**d**).

Echocardiography showed severe right atrial and ventricular dilation (Figure 2a,b) associated with right ventricular hypokinesia. Flattening of the interventricular septum was present and the internal dimension of the left ventricle was reduced. Moderate to severe tricuspid regurgitation was evident (Figure 2c) with normal morphology of the valvular leaflets. The peak velocity of the tricuspid regurgitation was 2.9 m/s. The pulmonary artery was dilated, with a pulmonary trunk to aorta ratio of 1.42. The pulmonary artery systolic flow had a proto-systolic peak (Figure 2d). Mild pericardial and pleural effusion and moderate abdominal effusion were evident. The analysis of the abdominal effusion indicated a modified transudate. All these findings were consistent with severe pre-capillary PH and R-CHF. The presence of congenital heart diseases was verified and excluded by two-dimensional and Doppler echocardiography.

**Figure 3:**
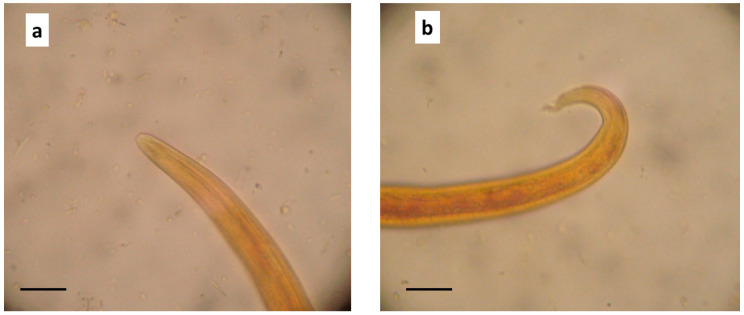
*Aelurostrongylus abstrusus* first stage larva (L1) in the Baermann examination of the kitten faecal sample. (**a**) Anterior end, (**b**) posterior end showing the S-shaped tail. 400x, (scale bar = 20 μm).

Several nematode L1s were found in the sediment of the Baermann test (about 20 L1/microscopic field at 40x, and about 4260 L1 per gram of faeces, LPG). In the microscopic examination, L1s were 360–415 μm in length and about 18–19 μm in width, showed a rounded head with a terminal opening (Figure 3a), and an S-shaped tail with distinct knob-like or small finger-like projections at the tip of cuticular spines (Figure 3b). For all these features, they were identified as *A. abstrusus* L1 [26].

Based on the clinical, parasitological, and molecular results, a parasitic bronchopneumonia caused by *A. abstrusus* infection, with secondary severe PH and R-CHF, was diagnosed.

### 3.2. Post-Mortem Examination

**Figure 4:**
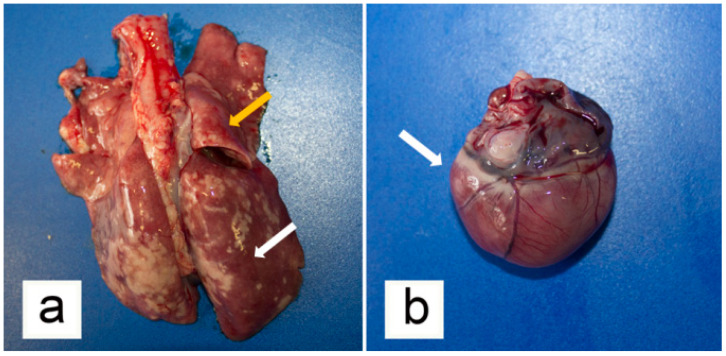
Gross pathological findings from the examined kitten. (**a**) Lungs showed diffuse hyperaemia (orange arrow) with lobular areas of consolidation (white arrow) and sparse whitish, slightly raised nodules. (**b**) Marked right-sided cardiac enlargement was evident (white arrow).

At necropsy, 50 mL of blood-tinged serous transudate was collected from the peritoneal cavity, and the liver surface was markedly covered with thin fibrin deposits. Other organs in the abdominal cavity were normal at gross examination. A total of 20 ml of blood-tinged transudate fluid was collected from the thoracic cavity and both lungs showed lobar areas of reddish consolidation, as well as sparse subpleural soft to firm greyish/whitish slightly raised nodules (Figure 4a). Pulmonary oedema and multifocal emphysema were present at the periphery of these lesions. On the cut surface of lung parenchyma, marked oedema, thickening of bronchiolar walls, and the presence of catarrhal exudate in the bronchial lumen were observed. No adult nematodes were macroscopically visible in the trachea, bronchi, and bronchioles lumen. Tracheobronchial lymph nodes were moderately enlarged. A small amount of transudate fluid was detected in the pericardial sac. The heart weighted 12 g and marked enlargement of the right atrial and ventricular cavities was evident (Figure 4b). Macroscopic examination of encephalon revealed congestion of blood vessels in the meninges.

At the examination of the post mortem lung lavage fluid, a countless number of L1 were evidenced. Molecular analysis of all biological samples taken from the kitten, i.e., lung lavage liquid, the Baermann sediment, and the faecal sample, confirmed the microscopic identification of the parasite as *A. abstrusus*.

**Figure 5:**
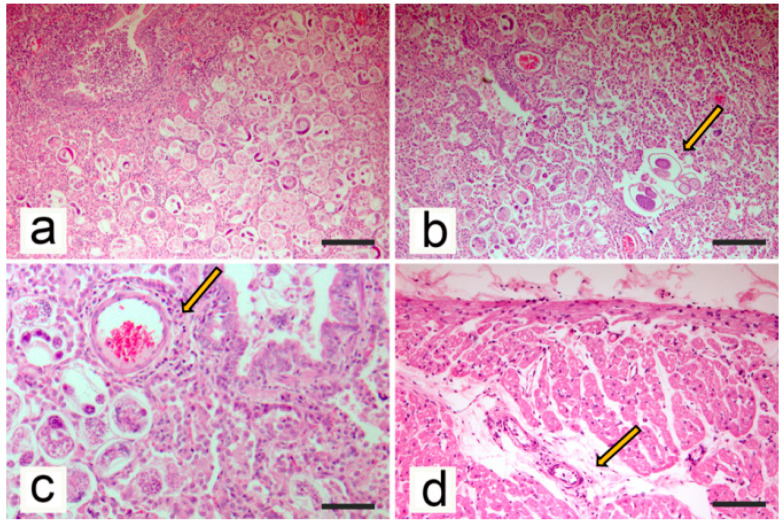
Histopathological findings from the examined kitten. (**a**) Lung. Alveolar lumen filled with eggs and larvae (Haematoxylin-Eosin stain, bar = 100 μm). (**b**) Lung. Scattered adult *Aelurostrongylus abstrusus* (arrow) were present within the inflammatory tissue, where the alveoli were filled by morulated eggs (Haematoxylin-Eosin stain, bar = 100 μm). (**c**) Lung. Absence of smooth muscle hypertrophy of arteriolar wall and presence of mild inflammatory cell infiltrate in the periarteriolar area (arrow; Haematoxylin-Eosin stain, bar = 50 μm). (**d**) Heart. Myocardial oedema with myocardiocyte spacing and presence of mild inflammatory infiltrates located in the periarteriolar areas (Haematoxylin-Eosin stain, bar = 100 μm).

Histopathologic examination revealed marked liver congestion with the accumulation of red blood cells in the centrilobular areas without acute swelling or necrosis of hepatocytes. Fibrin deposits were evident on the liver capsule in the absence of inflammatory infiltrates. In large areas of pulmonary parenchyma, alveolar lumen was filled by thin-walled morulated eggs and larvae accompanied by a diffuse thickening of the alveolar walls due to the presence of mixed inflammatory cell infiltrates constituted by eosinophils, neutrophils, and macrophages (Figure 5a), whereas granulomatous inflammation was rarely observed. Inflammatory lesions were mostly located in the peribronchial area. Alveolar capillary congestion and alveolar lumen dilatation were detected at the periphery of peribronchial areas. Cross-sections of adult nematodes (from 55 to 75 μm in diameter) were detected within the inflammatory infiltrates (Figure 5b). Inflammatory infiltrates were detected in the wall of larger and smaller bronchi and bronchioles, including lymphocytes and plasma cells. A mild hyperplasia of the bronchial-associated lymphoid tissue and the peribronchial glands was evident, in association with mucus and inflammatory cells in the bronchial lumina. The smooth muscles of bronchiolar walls and alveolar ducts were slightly hypertrophied. Pulmonary artery changes included the presence of mild inflammatory cell infiltrates located in the subintimal areas, aggregated into a cluster of periarteriolar areas (Figure 5c). Thickening of the smooth muscle of the arterial wall was not evident. Reactive lymphoid hyperplasia was detected in the tracheobronchial lymph nodes. Histopathologic examination of the heart samples revealed myocardial oedema with myocardiocyte spacing and arteriolar changes, characterized by the presence of mild inflammatory infiltrates located in the periarteriolar areas and constituted by mononuclear cells (Figure 5d). Significant histopathologic changes were not detected in the other examined tissues.

## 4. Discussion

Cat aelurostrongylosis may be subclinical, acute, or chronic, but it causes fatal pneumonia very rarely [1,10,28]. Most *A. abstrusus*-infected cats are subclinical or show a chronic cough with gradually increasing tachypnoea and respiratory distress, or other respiratory signs, as wheezing, sneezing, nasal discharge, frequently associated to apathy, anorexia, and fever [10,11,29,30]. Some kittens may develop more severe clinical forms than adult cats, probably due to a quicker occlusion of the airways by *A. abstrusus* due to the smaller diameter of the bronchi and the concomitant inflammation [31]. This happens more frequently in troglostrongylosis of kittens due to the size of *T. brevior* adults [1], as *A. abstrusus* is a tiny nematode, thus only highly parasitic burdens may occlude the airways of cats. In fact, lung damages in aelurostrongylosis are rather caused by the egg deposition with consequent pulmonary inflammation [12].

The present kitten presented PH. Based on the veterinary literature, the main differential diagnosis for PH in young cats includes congenital heart diseases (patent ductus arteriosus, supravalvular mitral stenosis, cor triatriatum sinister, atrioventricular septal defect) [14,15,16,20], lungworm diseases [21,22], and upper airway obstruction [19]. Differently from dogs, cats affected by heartworm or lungworm disease rarely develop PH, even if presenting significant pulmonary parenchymal and arterial damage [32,33]. Nevertheless, reversible PH with right ventricular enlargement and large systolic tricuspid regurgitation associated with *A. abstrusus* infection have been previously recorded in one ten-week-old kitten [22]. In this latter case, only moderate right atrial enlargement was described, with no evidence of right-sided congestive heart failure. Conversely, in the clinical case here in described, severe right atrial enlargement was present, and pericardial, pleural, and abdominal effusions were compatible with right-sided congestive heart failure.

No echocardiographic evidence of PH was reported in a study on cats infected by *A. abstrusus* [32]. However, the median age of the included population was 24 months, with the youngest cat ageing 8 months. It can be thus argued that age is a critical factor in triggering the onset of PH, in consideration of the age of the kitten with PH which was described in the Netherlands [22] and of the kitten presented here. Nevertheless, reversible PH with right ventricular enlargement and large systolic tricuspid regurgitation associated with *A. abstrusus* infection have been previously recorded in one ten-week-old kitten [19]. In this latter case, only moderate right atrial enlargement was described, with no evidence of R-CHF. Conversely, in the present clinical case, the evidence of severe right atrial and ventricular dilation, the flattening of the interventricular septum, the reduced left ventricular dimension, and the pulmonary artery enlargement were consistent with severe PH [14,15,16,17,20]. Additional Doppler signs of PH were the increased velocity of the tricuspid regurgitation and the proto-systolic peak of the pulmonic flow [14,15,16,17,20]. The pericardial, pleural, and abdominal effusions were compatible with R-CHF. A possible higher tricuspid regurgitation peak velocity could have been expected in a case of PH causing R-CHF. However, the presence of right ventricular systolic disfunction, the high right atrial pressure, and possible technical factors such as high frequency transducers and suboptimal alignment, could have produced a lower peak velocity. Differential diagnosis for R-CHF in domestic cats include congenital heart diseases (mainly tricuspid valve dysplasia, tetralogy of Fallot, endocardial cushion defect, patent ductus arteriosus, double chambered right ventricle) [34,35,36] and acquired cardiomyopathies (mainly restrictive cardiomyopathy and arrhythmogenic right ventricular cardiomyopathy) [37,38]. To the authors’ knowledge, this the first report of severe PH and R-CHF of parasitic origin in a kitten.

Other than age, parasite load and other unknown risk factors may play a central role in the development of clinical signs, including PH, in cats naturally infected with *A. abstrusus* [2]. Indeed, in addition to the direct damages caused by adult nematodes to the lung parenchyma, eggs, larvae, and inflammatory exudate induce bronchiolar muscular hypertrophy and hyperplasia of the smooth muscle of the pulmonary arteries, which may gradually obstruct the bronchiolar system and cause PH [12,39,40]. Pulmonary vasoconstriction elicited by mast cells and histamine release, promoting pulmonary vascular resistance, was also hypothesized [2,22].

The LPG found in the kitten faeces in the Baermann test, i.e., about 4260 LPG, can be considered a high value. In fact, a mean number of 1198 LPG was counted in the majority of symptomatic cats showing severe radiographic changes in a previous study [41], although cats experimentally infected with a high dose of *A. abstrusus* L3 and showing severe lung lesions may shed a variable number of L1s, down to 687 [10]. Although the LPG number counted in the Baermann test in the kitten herein examined may be considered consistent with the very high number of L1 found in the lung, in general there is no a constant concordance between number of L1 shed by infected animals and the degree of lung damages and severity of clinical signs. Additionally, the period elapsed between the death of the kitten and the parasitological analysis of the lungs could have allowed the eggs present in the examined lung tissue to hatch, resulting in the detection of an extremely high L1 number.

Interestingly, *A. abstrusus* infection is a frequent finding in cats deceased during anaesthesia [42]. Sedation or anaesthesia may reduce a cat’s ability to compensate for diminished gas exchange surface area, compromising lung perfusion and ventilation, which can lead to hypoxia, systemic hypotension, and cardiovascular arrest. For these reasons, pre-anaesthesia parasitological screening for lungworms is strongly suggested [42]. This is particularly true for kittens, given that age could play a role in the onset of PH impacting on cardiorespiratory functions.

Pathologic findings in the clinical case showed in this study are in line with previous studies [10,13,22]. Regarding changes in the pulmonary arteries, hyperplasia, and hypertrophy of the smooth muscle cells of the media and intimal proliferation are commonly reported, resulting in narrowing of the lumen of pulmonary vasculature [12]. However, in the herein described case, no significant changes in the media and intima were noted. Based on the severe pulmonary parenchymal lesions, it is possible that PH occurred secondarily to lung damages and hypoxemia. Similar findings were already reported in a kitten with severe pulmonary parenchymal disease [22]. Diffuse alveolar disease can lead to impaired oxygen exchange in the lungs [43]. In addition, *A. abstrusus* may cause prolonged vasoconstriction mediated by mast cells and histamine release, triggering increased pulmonary vascular resistance and PH [44].

Irreversible pulmonary hypertension was recently reported in a cat infected with the lungworm *T. brevior* [21]. Importantly, this nematode is more pathogenic than *A. abstrusus* and it is known that kittens often develop severe or fatal infections by *T. brevior* rather than cats harbouring *A. abstrusus* [9,45]. Thus, a reliable parasitological diagnostic work-up is important to differentiate these lungworms towards appropriate and efficacious anthelmintic treatments [11]. The detection of L1 in the Baermann test and a thorough microscopic analysis are crucial. It has been recently claimed that the length of *A. abstrusus* and *T. brevior* L1s is distinctively different, i.e., ~360–415 and ~300–357 μm, respectively [26]. Nonetheless, L1s of both species with length outside of these ranges have also been described [1,4]. Therefore, other features should be examined to achieve a definitive identification. In particular, *A. abstrusus* L1s have a rounded head with a terminal oral opening and an S-shaped tail with a distinct knob-like or small finger-like projections at the tip of cuticular spines, while *T. brevior* L1s have a pointed head and a subterminal oral opening, with a tail tapering to the extremity and showing a deep dorsal notch and a shallower ventral one near their tip [26]. DNA-based approaches are advisable in doubtful cases [27].

## 5. Conclusions

This is the first report of fatal severe PH and right-sided congestive heart failure in a kitten infected with *A. abstrusus*. Thus, other than in clinical cases of respiratory distress, *A. abstrusus* should be included in the differentials in the case of PH and right-sided congestive heart failure, especially in kittens and young cats. This is of importance, as cases of PH in association with *A. abstrusus* infection up to now have been reported only sporadically.
